# Supplementary material for: GABAA Receptor-Mediated Currents and Hormone mRNAs in Cells Expressing More Than One Hormone Transcript in Intact Human Pancreatic Islets
Source: Int J Mol Sci. 2020 Jan 17;21(2):600. doi: 10.3390/ijms21020600 (PMC7013858; doi:10.3390/ijms21020600)
Supplement: Supplementary file 1 [file ijms-21-00600-s001.pdf]

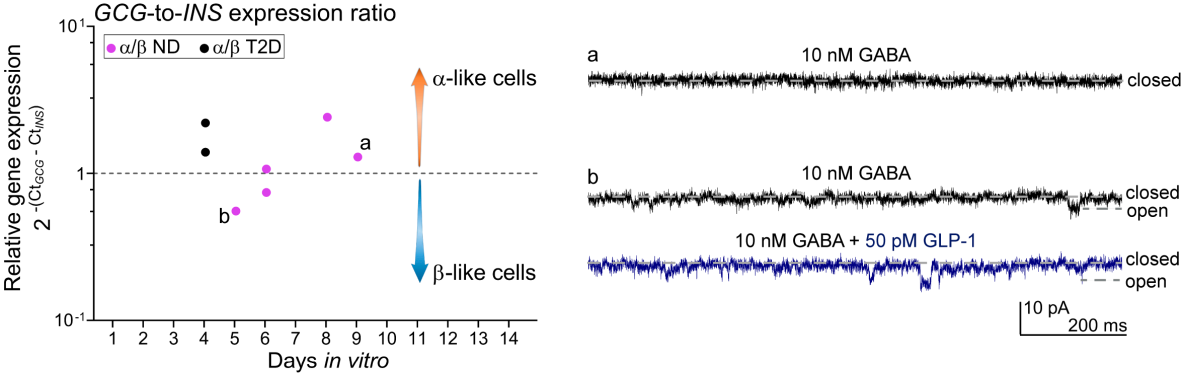

Supplementary Figure S1. Ratios of hormone mRNA expressions *GCG*/*INS* close to 1 in the mixed-identity α/β cells and representative $iGABA_A R$-mediated currents in such cells. The cells with *GCG*/*INS* ≥ 1 either did not have single-channel $iGABA_A R$ activity or it was very low, consistent with α-like cell phenotype (a). In contrast, in a cell with *GCG*/*INS* ~ 0.55 (≤ 1) the $iGABA_A R$ activity was remarkably higher and it was potentiated by application of 50 pM GLP-1 that resembles the behavior of the β-like cells (b). The GABA in the concentration 10 nM was added to the extracellular solution when the recordings from the cells a and b were done; 50 pM GLP-1 was further added to the extracellular solution in order to examine the potentiation of $iGABA_A Rs$ via the activation of GLP-1 receptor (b). Closed and open states of the single channels are denoted by corresponding dash lines on the recordings. The glucose concentration in all experiments was 20 mM. ND, no diabetes; T2D, type 2 diabetes.
